# Gender Differences after Transcatheter Aortic Valve Replacement (TAVR): Insights from the Italian Clinical Service Project

**DOI:** 10.3390/jcdd8090114

**Published:** 2021-09-15

**Authors:** Andrea Denegri, Michele Romano, Anna Sonia Petronio, Marco Angelillis, Cristina Giannini, Claudia Fiorina, Luca Branca, Marco Barbanti, Giuliano Costa, Nedy Brambilla, Valentina Mantovani, Matteo Montorfano, Luca Ferri, Giuseppe Bruschi, Bruno Merlanti, Bernhard Reimers, Carlo Pivato, Arnaldo Poli, Carmine Musto, Massimo Fineschi, Diego Maffeo, Carlo Trani, Flavio Airoldi, Corrado Lettieri

**Affiliations:** 1 Cardiology Division, Department of Biomedical, Metabolic and Neural Sciences, University of Modena and Reggio Emilia, Policlinico di Modena, 41125 Modena, Italy; 2 Division of Cardiology, Ospedale Carlo Poma, ASST Mantova, 46100 Mantova, Italy; michele.romano@asst-mantova.it (M.R.); lettieri.corrado@asst-mantova.it (C.L.); 3 Cardiothoracic and Vascular Department, Azienda Ospedaliero-Universitaria Pisana, 56126 Pisa, Italy; a.petronio@ao-pisa.toscana.it (A.S.P.); ma.angelillis@ao-pisa.toscana.it (M.A.); cr.giannini@ao-pisa.toscana.it (C.G.); 4 Cardiothoracic Department, Spedali Civili Brescia, 25123 Brescia, Italy; claudia-fiorina@asst-spedalicivili.it (C.F.); luca.branca@asst-spedalicivili.it (L.B.); 5 Division of Cardiology, Policlinico-Vittorio Emanuele Hospital University of Catania, 95124 Catania, Italy; mbarbanti83@gmail.com (M.B.); giuliano.costa@policlinico.unict.it (G.C.); 6 Department of Cardiology, Policlinico San Donato, 20097 Milano, Italy; nedy.brambilla@grupposandonato.it (N.B.); valentina.mantovani@grupposandonato.it (V.M.); 7 Interventional Cardiology Division, Cardio-Thoracic-Vascular Department, San Raffaele Scientific Institute, 20132 Milano, Italy; montorfano.matteo@hsr.it (M.M.); ferri.luca@hsr.it (L.F.); 8 Department of Cardiology, ASST Grande Ospedale Metropolitano Niguarda, 20162 Milan, Italy; giuseppe.bruschi@ospedaleniguarda.it (G.B.); bruno.merlanti@ospedaleniguarda.it (B.M.); 9 Department of Cardiology, Istituto Clinico Humanitas, 20089 Milan, Italy; bernhard.reimers@humanitas.it (B.R.); carlo.pivato@humanitas.it (C.P.); 10 Interventional Cardiology Unit, ASST Ovest Milanese, Legnano Hospital, 20025 Milan, Italy; arnaldo.poli@asst-ovestmi.it; 11 Division of Cardiology, S. Camillo-Forlanini Hospital, 00152 Roma, Italy; cmusto@hotmail.it; 12 Department of Internal, Cardiovascular and Geriatric Medicine, Azienda Ospedaliera Universitaria Senese, 53100 Siena, Italy; massimofineschi67@gmail.com; 13 Department of Interventional Cardiology, Fondazione Poliambulanza, 25124 Brescia, Italy; diego.maffeo@poliambulanza.it; 14 Department of Cardiovascular and Thoracic Sciences, Fondazione Policlinico Universitario A. Gemelli IRCCS, 00168 Roma, Italy; carlo.trani@policlinicogemelli.it; 15 Cardiovascular Department, IRCCS MultiMedica Hospital, 20099 Milano, Italy; flavio.airoldi@multimedica.it

**Keywords:** aortic stenosis, TAVR, gender differences, sex differences, women, Medtronic, Corevalve, Evolut R, Evolut Pro, CV-outcome, mortality

## Abstract

Background: TAVR is a safe alternative to surgical aortic valve replacement (SAVR); however, sex-related differences are still debated. This research aimed to examine gender differences in a real-world transcatheter aortic valve replacement (TAVR) cohort. Methods: All-comer aortic stenosis (AS) patients undergoing TAVR with a Medtronic valve across 19 Italian sites were prospectively included in the Italian Clinical Service Project (NCT01007474) between 2007 and 2019. The primary endpoint was 1-year mortality. We also investigated 3-year mortality, and ischemic and hemorrhagic endpoints, and we performed a propensity score matching to assemble patients with similar baseline characteristics. Results: Out of 3821 patients, 2149 (56.2%) women were enrolled. Compared with men, women were older (83 ± 6 vs. 81 ± 6 years, *p* < 0.001), more likely to present severe renal impairment (GFR ≤ 30 mL/min, 26.3% vs. 16.3%, *p* < 0.001) but had less previous cardiovascular events (all *p* < 0.001), with a higher mean Society of Thoracic Surgeons (STS) score (7.8% ± 7.1% vs. 7.2 ± 7.5, *p* < 0.001) and a greater mean aortic gradient (52.4 ± 15.3 vs. 47.3 ± 12.8 mmHg, *p* < 0.001). Transfemoral TAVR was performed more frequently in women (87.2% vs. 82.1%, *p* < 0.001), with a higher rate of major vascular complications and life-threatening bleeding (3.9% vs. 2.4%, *p* = 0.012 and 2.5% vs. 1.4%, *p* = 0.024). One-year mortality differed between female and male (11.5% vs. 15.0%, *p* = 0.002), and this difference persisted after adjustment for significant confounding variables (Adj.HR1yr 1.47, 95%IC 1.18–1.82, *p* < 0.001). Three-year mortality was also significantly lower in women compared with men (19.8% vs. 24.9%, *p* < 0.001) even after adjustment for age, STS score, eGFR, diabetes and severe COPD (Adj.HR3yr 1.42, 95%IC 1.21–1.68, *p* < 0.001). These results were confirmed in 689 pairs after propensity score matching. Conclusion: Despite higher rates of peri-procedural complications, women presented better survival than men. This better adaptive response to TAVR may be driven by sex-specific factors.

## 1. Introduction

Transcatheter aortic valve replacement (TAVR) has been proven to be an effective treatment for inoperable, high-, intermediate-, and even low-risk symptomatic severe aortic stenosis (AS) [1,2,3,4,5,6,7]. Women are usually underrepresented in most clinical trials, except for TAVR studies, in which they account up to 50% of patient population [8]. Despite the higher rate of periprocedural complications, the female sex has been related to better long-term survival, particularly in case of trans-femoral TAVR [9,10]. Moreover, despite the fact that several studies have explored female-specific factors in TAVR and their association with protection from future cardiovascular events, the results are still debated [11,12]. The understanding of these risk differences is essential to better individualize TAVR treatment and to investigate whether female sex–specific characteristics contribute to TAVR outcomes. This analysis aims to report clinical outcomes of women undergoing TAVR across Italy over the last decade.

## 2. Materials and Methods

From June 2007 to July 2019, all consecutive patients with severe aortic stenosis undergoing TAVR with the CoreValve, Evolut R and Evolut Pro (Medtronic Inc., Minneapolis, MN, USA) devices admitted to the participating Italian centers were prospectively included in the One Hospital Clinical ServiceProject. This is a nation-based clinical data repository and medical care quality improvement project aimed to describe and improve the use of implantable devices in clinical practice (clinicaltrials.gov NCT01007474). The project was approved by each site’s institutional review board or medical director and conforms to the principles outlined in the Declaration of Helsinki. Each patient signed an informed consent for data collection and analysis. Eligibility for TAVR was established at each center, based on the consensus of the local Heart Team.

### 2.1. Eligibility Criteria

All the patients included in the Clinical Service Project were deemed eligible with the exception of those undergoing TAVR for a failed surgical aortic bioprosthesis and patients with bicuspid valves. The main inclusion criteria were the following: Severe AS determined by echocardiography with a mean gradient >40 mm Hg or peak jet velocity >4.0 m/s and an aortic valve area <0.8 cm^2^ or aortic valve area index <0.5 cm^2^/m^2^. The main exclusion criteria were: (1) hemodynamic instability; (2) active endocarditis or sepsis within 6 months before the TAVR procedure; (3) life expectation <12 months.

### 2.2. TAVR Procedure and Clinical Follow-Up

Evaluation of medical history, transthoracic or transesophageal echocardiography, as well as thoracic computed tomography, was performed to assess AS. Procedural decisions regarding TAVR access and device were at the discretion of the treating physicians. Patient follow-up was conducted by a standardized phone contact or clinic visit at 1 month, 12 months and then yearly after TAVR to record clinical status and occurrence of adverse events.

### 2.3. Endpoints and Definitions

The primary endpoint was 1-year all-cause mortality. We also investigated a composite of major adverse cardiovascular and cerebrovascular events (MACCE) including all-cause mortality, stroke and myocardial infarction as well as 3-year mortality. Endpoints were adjudicated using the standardized VARC-2 criteria [13].

### 2.4. Statistical Analysis

Descriptive statistics were used to summarize patient characteristics. This includes mean and standard deviation, minimum, maximum and median with the interquartile range [IQR] for continuous variables, counts and percentages for categorical variables. Comparisons between groups were performed using Wilcoxon’s test for continuous variables, while comparisons of categorical variables were performed by means of the Chi-square test or Fisher exact test for extreme proportions, as appropriate. Statistical tests were based on a two-sided significance level of 0.05. The follow-up duration (months) will be computed from the date of the implantation to the date of the last available follow-up or date of event or date of exit (not lost to follow-up) from ClinicalService. The analyses of time-to-first event were described using Kaplan–Meier curves and compared between the groups with the log-rank test. To find predictors for events, a Cox regression was imputed for both univariate and multivariate analyses. A propensity score method has been used to adjust for differences in baseline characteristics between the female and male groups. The propensity scores for each patient were calculated by using a logistic regression model that included the following variables: age, hypertension, diabetes, CAD, peripheral vascular disease, prior stroke, GFR, STSScore, femoral access and LVEF. The SAS software version 9.4 (SAS Institute Inc, NC, USA) was used to perform the analysis.

## 3. Results

Baseline characteristics are resumed in Table 1.

Out of 3821 patients, 2149 (56.2%) women were enrolled. Compared with men, women were older (83 ± 6 vs. 81 ± 6, *p* < 0.001), with higher prevalence of severe renal impairment (GFR ≤ 30 mL/min, 26.3% vs. 16.3%, *p* < 0.001). Mean Society of Thoracic Surgeons (STS) score was higher in women (7.8% ± 7.1 vs. 7.2% ± 7.5, *p* < 0.001). Women presented higher mean aortic gradients (52.4 mmHg ± 15.3 vs. 47.3 mmHg ± 12.8, *p* < 0.001) but a better left ventricular ejection fraction (LVEF, 54.3% ± 11.2 vs. 49.7% ± 12.7, *p* < 0.001). TAVR was performed more frequently via transfemoral access in women (87.2% vs. 82.1%, *p* < 0.001). The Corevalve self-expanding valve was the most implanted device (56.9% vs. 43.1%, *p* < 0.001). The most common prothesis size was 26 mm for women (63.6% vs. 13.2%, *p* < 0.001) and 29 mm for men (62.9% vs. 28.1%, *p* < 0.001). Women presented the highest procedural success (97.5% vs. 95.8%, *p* = 0.004). All peri-procedural complications are listed in Table 2. Major vascular complications and life-threatening bleeding have occurred more frequently in women (3.9% vs. 2.4%, *p* = 0.012 and 2.5% vs. 1.4%, *p* = 0.024, respectively), while permanent PM implantation was performed more frequently in men (26.1% vs. 21.2%, *p* < 0.001).

Women presented a numerical increase in peri-procedural cerebrovascular accident (2.6% vs. 1.8%, *p* = n.s.). No statistical differences were found in terms of peri-procedural death, myocardial infarction (MI), acute kidney injury (AKI) or unplanned interventions (UI). Women, moreover, presented the best results in terms of LVEF improvement and para-valvular leak occurrence, as reported in Table 3.

**Figure 1:**
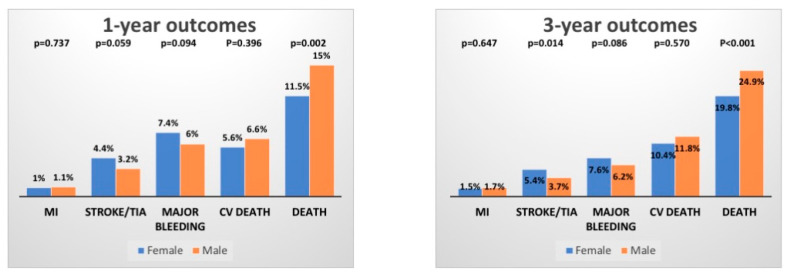
Cardiovascular outcomes after TAVR.

At 1-year follow-up, the primary endpoint occurred in 11.5% of females vs. 15.0% males (*p* = 0.002). Similar results were found considering 3-year mortality (19.8% vs. 24.9%, *p* < 0.001) as shown in Figure 1.

**Figure 2:**
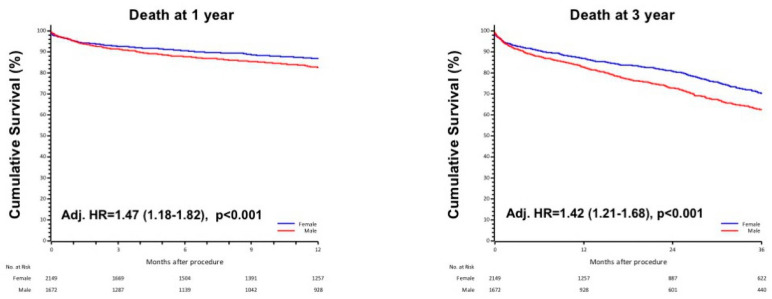
The 1-year and 3-year death in male and female, adjusted respectively for age, STS score, eGFR, diabetes and for age, STS score, eGFR, diabetes and severe COPD.

KM curves showed a higher all-cause mortality risk at 1-year in men compared with women (HR1yr 1.32, 95%IC 1.10–1.57, *p* = 0.002), which persisted after adjustment for significant confounding variables by a stepwise regression model (Adj. HR1yr 1.47, 95%IC 1.18–1.82, *p* < 0.001). Similar findings were reported when considering 3-year mortality (HR3yr 1.34, 95%IC 1.17–1.53, *p* < 0.001), even after adjustment for age, STS score, diabetes, eGFR and severe COPD (Adj. HR3yr 1.42, 95%IC 1.21–1.68, *p* < 0.001). These results are shown in Figure 2.

**Figure 3:**
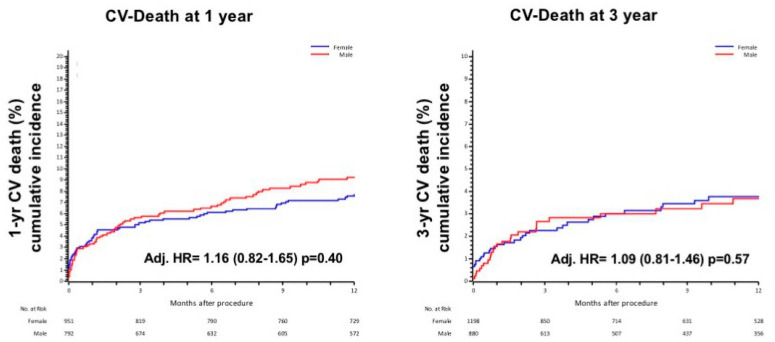
1-year and 3 year CV death in male and female, adjusted for age, STS, previous MI and CAD.

No statistical difference was found in cardiovascular (CV) death at 1-year (Adj HR = 1.16 (0.82–1.65) *p* = 0.40) and 3-year (Adj. HR = 1.09 (0.81–1.46) *p* = 0.57, Figure 3).

**Figure 4:**
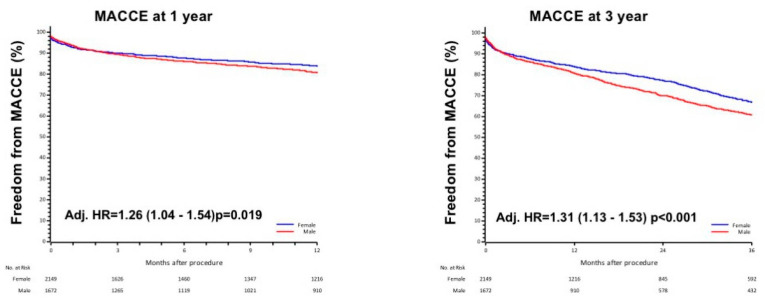
Composite endpoint (MACCE) at 1- and 3-year in male and female, adjusted respectively for age, STS score, eGFR, diabetes and for age, STS score, eGFR, diabetes and severe COPD.

Considering the composite endpoint of MACCE, men presented a persistently 30% higher risk compared with women even after adjustment for baseline confounding variables (Adj. HR1yr = 1.26 (1.04–1.54) *p* = 0.019 and Adj. HR3yr = 1.31 (1.13–1.53) *p* < 0.001, Figure 4).

**Figure 5:**
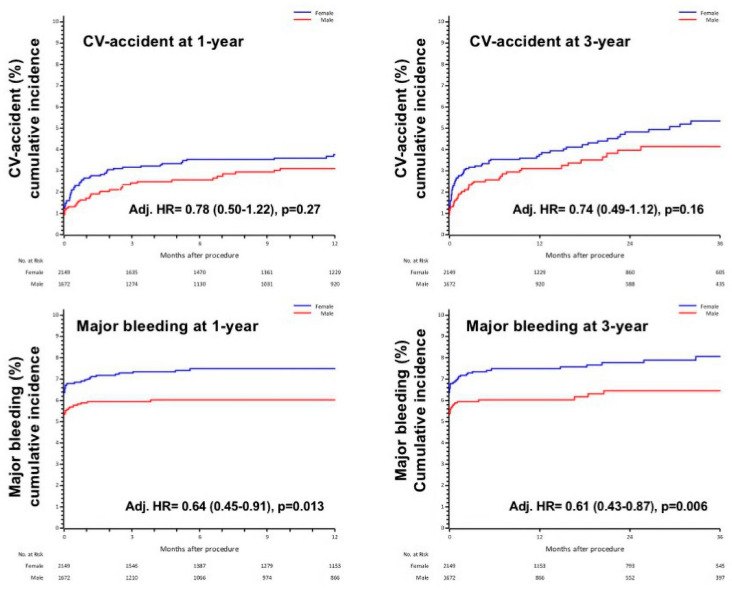
1-year and 3-year stroke and bleeding in male and female, adjusted respectively for age and eGFR, and for age, CAD, MI and PAD.

Analyzing each single component of the composite endpoint, women presented a numerical increase in cerebrovascular accident, with 1- and 3-year incidence of 4.4% vs. 3.2%, *p* = 0.059 and 5.4% vs. 3.7%, *p* = 0.014, respectively. Major bleeding accounted in female sex for 7.4% vs. 6.0%, *p* = 0.094 at 1-year and 7.6% vs. 6.2%, *p* = 0.086 at 3-year. Men presented lower incidence of major bleeding after adjustment for confounding factors such as age, eGFR, CAD, MI and PAD (Adj. HR1yr 0.64 (0.45–0.91) *p* = 0.013 and Adj. HR3yr 0.61 (0.43–0.87) *p* = 0.006, Figure 5).

Beside a decrease in absolute rates of stroke and major bleedings when considering the first five years of observation compared to the last five, the only statistical difference is related to major bleeding in the first five years of observation; no other statistical differences between sexes were reported (Supplementary Figure S1).

After propensity score matching, we compared 689 women and men. Women presented lower rate of mortality at 1- and 3-year (9.7% vs. 14.8%, *p* = 0.004 and 18.0% vs. 24.1%, *p* = 0.006) compared with men with a numerical increase in major bleeding (6.2% vs. 4.4%, *p* = 0.118 and 6.8% vs. 4.5%, *p* = 0.062) and stroke (3.0% vs. 1.7%, *p* = 0.113 and 3.8% vs. 2.0%, *p* = 0.054), and this was confirmed after adjustment for confounding variables, highlighting better survival for women (Supplementary Figure S2).

## 4. Discussion

This multicenter, prospective, real-world cohort of TAVR patients highlighted the following points: (1) a significant survival benefit of women undergoing TAVR for severe AS compared with males, clearly statistically significant at 1- and 3-year; (2) a significant reduced rate of MACCE according to female sex at 1- and 3-year, although a higher numerical increase in stroke/TIA in women; (3) diabetes mellitus (DM) and peripheral artery disease (PAD), beyond male sex, remained significant predictors of death and major vascular complications, respectively; and (4) a preserved renal function, defined by eGFR > 60 mL/min, represents one of the most important predictors of survival.

Despite higher rates of peri-procedural complications such as major bleeding and vascular complications, as reported in previous analyses [14], women have been already related to better 1-year survival after TAVR compared with men [8,10,11,15,16]. Our results confirmed this trend in the long term, with a more pronounced reduction in mortality rates at 3-year. This may partially be explained by the different risk factor profile, with lower incidence of coronary artery disease (CAD), PAD and DM, beyond a higher baseline LVEF [17]. Female sex has been indeed associated with a different response to pressure overload, with a more concentric left ventricular hypertrophy rather than the more eccentric one observed in men, which may lead to ventricular dilation, fibrosis, a decreased ejection fraction and development of heart failure, as already reported in previous studies [18,19]. Moreover, it has been demonstrated that women present a significant improvement of LVEF after TAVR [20], which may account for superior survival. Our 1-year death rate (11.5%) is in line with that reported by PARTNER II trial (12.3%), confirmed by a recent meta-analysis [2,21]. Women of our cohort have been associated with better survival also at 3-year, as similarly reported in a recent publication [22]. In our analysis, mortality rates did not differ when considering CV death, and this supports the better outcome of women compared with men, despite a higher rate of peri-procedural complications, including major bleeding. Recent data have demonstrated that this favorable gender-driven outcome seems to decrease with contemporary TAVR, mainly due to technological improvement and availability of different valve sizing, observed also in our research but not related to outcome changes [23]. This finding is confirmed in a recent analysis that demonstrated how gender-related disparities did not translate into a significant difference in clinical outcomes in men and women [24]. In our analysis, besides a decrease in absolute peri-procedural complications rate, the only statistical difference was observed between genders when considering major bleeding during the first five years of observation. The impact of gender on long-term outcomes after TAVR is, indeed, still debated [25].

Cerebrovascular accident in our cohort (4.4%) is mildly lower compared with that (5.4%) reported in the SURTAVI (Surgical Replacement and Transcatheter Aortic Valve Implantation) [26], and this may be related to baseline patient characteristics, which presented less comorbidities, even if it is well known that women have been shown to be at higher risks of stroke than men [27].

DM is an established component of pre-operative risk assessment in AS patients and was an independent predictor of mortality also in our research. Although diabetic patients presented an overall lower survival than those without diabetes, insulin-treated diabetes was not an independent risk factor for higher mortality in a recent analysis [28]. Among female patients of the WIN-TAVI international registry, DM was not associated with increased mortality after TAVR [29].

PAD is a frequent comorbidity of AS patients undergoing TAVR and has been significantly associated with increased rates of major vascular complications as well as long-term mortality [30]. Recent data suggested the role of PAD in independently predicting early and long-term mortality [31]. In our analysis, PAD was an independent predictor of major vascular complications, which occurred significantly more frequently in women compared with men. This may be partially explained by potentially lower vascular dimension and higher BMI, although trans-femoral access has been demonstrated to be associated with the best long-term results [32]. A recent study confirmed that women and men present similar rates of short-term mortality with transfemoral access, although the female sex is burdened by higher rates of peri-procedural complications after TAVR [33]. A surgical approach may be used to minimize the rate of major vascular complications, particularly in high-risk bleeding patients, as reported by previous studies [34].

CKD has been associated with higher mortality and morbidity in women undergoing TAVR [35], and, in our analysis, it was an independent predictor of life-threatening bleeding. A recent meta-analysis confirmed the higher risk of mortality and procedural complications in patients with severe CKD [36]. About one-fourth of female patients in our analysis presented a severe impairment of renal function that is frequently related to lower levels of hemoglobin. According to recent data, severe anemia in women undergoing TAVR has been associated with increased rates of VARC-2 efficacy endpoint, including stroke [37]. Although it is well known that TAVR reduces the risk of bleeding compared with SAVR [38], a higher incidence of anemia may partially explain the higher rate of major bleeding, also in the long term, affecting women undergoing TAVR.

Over the last decade, several technical improvements have been implemented, and this was possible also due to new-generation valves, although most of the patients enrolled in our study were treated with first-generation device. Moreover, safety and efficacy of new-generation self-expanding Medtronic Evolut R with the former-generation Corevalve are comparable [39]. A recent analysis has finally investigated efficacy and safety of the Evolut PRO and the Evolut R valve in a real-world setting, confirming comparable outcomes between the two systems [40].

The propensity score matched analysis, finally, confirmed better survival for women compared with men, once adjusted for the intergroup differences in baseline characteristics. This better response to TAVR may be driven by sex-specific factors.

### Predictors of Long-Term Outcomes

EuroSCORE I and EuroSCORE II are well-known predictors of procedural mortality with cardiac surgery [41,42] and have been associated with outcomes in women undergoing TAVR [4]. Similarly, STS has been described to be associated with procedural mortality with cardiac surgery, but its association with TAVR women has not been clarified. Of note is that components of risk calculators for cardiac surgery are not considered for TAVR [43]. Our findings also support a role for diabetes, PAD and COPD in predicting worse TAVR outcomes, emphasizing the need to stratify women’s TAVR risk [44]. Transfemoral access has been demonstrated to be the safest approach for TAVR, with lower rate of short- and long-term mortality compared with non-transfemoral access, and, despite a higher rate of peri-procedural complications in our cohort, it has been linked to better long-term outcomes especially in women [2].

## 5. Limitations

Our results are limited by several conditions. First of all, this a retrospective analysis of a real-world prospectively enrolled cohort of AS patients, without a randomized control arm. The possibility of selection bias reflected by sex differences in baseline characteristics and comorbidities cannot be completely ruled out, as men at baseline outnumbered females in terms of diabetes mellitus, CAD, COPD, dialysis, lower LVEF and higher logistic EuroSCORE, while females were older with higher percentage of arterial hypertension. The incidence of stroke may be underestimated due to a lack of systematic neurological evaluation after TAVR. The lack of systematic data collection regarding vessels’ characteristics, biomarkers and echocardiographic parameter values availability at follow-up represents another important limitation of our research.

## 6. Conclusions

Intermediate to high-risk women included in this multicenter, prospective, real-world cohort of AS patients experienced lower rate of 1- and 3-year mortality as well as 1- and 3-year MACCE. Diabetes is an independent predictor of mortality while a preserved renal function, defined by eGFR > 60 mL/min, represents one of the most important predictors of survival.

## Figures and Tables

**Table 1:** Baseline characteristics.

All Patients (*n* = 3821)			
	Female (*n* = 2149)	Male (*n* = 1672)	*p* Value
**Clinical Characteristics**			
Age	83 ± 6	81 ± 6	<0.001
Weight	65 ± 14	75 ± 12	<0.001
BMI	26 ± 5	26 ± 4	<0.001
Hypertension	84.5% (1807/2138)	81.3% (1351/1662)	0.008
Diabetes	27.7% (588/2123)	33.0% (546/1656)	<0.001
CAD	31.7% (651/2056)	49.8% (799/1604)	<0.001
Previous MI	10.8% (222/2058)	22.9% (370/1615)	<0.001
Previous PCI	21.2% (444/2094)	33.2% (543/1634)	<0.001
Previous CABG	5.9% (126/2149)	21.2% (355/1672)	<0.001
Previous Stroke	5.0% (76/1513)	7.7% (95/1227)	0.003
Previous PM	10.5% (215/2043)	16.2% (259/1594)	<0.001
PAD	16.2% (348/2149)	25.7% (430/1672)	<0.001
Severe COPD	14.0% (301/2149)	23.6% (395/1672)	<0.001
NYHA III/IV	77.4% (1544/1995)	76.8% (1201/1564)	n.s
Hemoglobin	11.6 ± 1.5	12.2 ± 1.8	<0.001
eGFR < 30 mL/min	26.3	16.3	<0.001
LogEuroscore	19.2 ± 13.3	19.8 ± 15.5	n.s
STS	7.8 ± 7.1	7.2 ± 7.5	<0.001
**ECG Features**			
LBBB	8.7% (186/2149)	8.5% (142/1672)	n.s
RBBB	5.1% (109/2149)	8.4% (140/1672)	<0.001
LAHB	7.7% (165/2149)	9.3% (156/1672)	n.s.
Any of above	19.4% (416/2149)	22.7% (379/1672)	0.012
**Echocardiographic Parameters**			
LVEF	54.3 ± 11.2	49.7 ± 12.7	<0.001
Mean aortic gradient	52.4 ± 15.3	47.3 ± 12.8	<0.001
Aortic regurgitation 2+	28.8% (534/1853)	29.1% (425/1459)	n.s.
Mitral regurgitation 2+	43.4% (855/1969)	37.4% (565/1510)	<0.001
sPAP < 60 mmHg	9.4% (163/1738)	8.2% (106/1293)	n.s.
**Procedural Characteristics**			
General anesthesia	24.5% (509/2076)	24.9% (402/1613)	n.s.
**Access**			
Femoral Subclavian Aortic Other	87.2% (1856/2129) 9.1% (194/2129) 3.6% (76/2129) 0.1% (3/2129)	82.1% (1363/1661) 14.1% (234/1661) 3.5% (58/1661) 0.4% (6/1661)	<0.001
**Prothesis Type**			
Corevalve Evolut Pro Evolut R	55.2% (1180/2137) 9.6% (206/2137) 35.1% (751/2137)	59.1% (983/1664) 6.1% (102/1664) 34.8% (579/1664)	< 0.001
Pre-dilation	69.1% (1216/1761)	70.1% (955/1363)	n.s.
**Prothesis Size**			
23 mm 26 mm 29 mm 31 mm 34 mm	6.5% (136/2096) 63.6% (1334/2096) 28.1% (589/2096) 0.8% (16/2096) 1.0% (21/2096)	0.9% (14/1631) 13.2% (215/1631) 62.9% (1026/1631) 11.0% (179/1631) 12.1% (197/1631)	<0.001
Post-dilation	25.6% (501/1954)	31.7% (485/1530)	<0.001
Procedural time	110.8 ± 51.9	113.8 ± 50.0	0.040
Fluoroscopy time	24.0 ± 15.9	26.2 ± 49.5	0.042
Contrast amount	165.0 ± 101.0	174.0 ± 87.2	<0.001
Procedural success	97.5% (2091/2145)	95.8% (1599/1669)	0.004

BMI = body mass index; CAD = coronary artery disease; MI = myocardial infarction; PCI = percutaneous coronary intervention; CABG = coronary artery by-pass graft; PM = pace maker; PAD = peripheral artery disease; COPD = chronic obstructive pulmonary disease; NYHA = New York heart association; GFR = glomerular filtration rate; STS = society of thoracic surgeons; LBBB = left bundle branch block; RBBB = right bundle branch block; LAHB = left anterior hemi-block; AF = atrial fibrillation; LVEF = left ventricular ejection fraction; PAPs = pulmonary artery pressures.

**Table 2:** Peri-procedural complications.

All Patients (*n* = 3821)			
	Female (*n* = 2149)	Male (*n* = 1672)	*p* Value
Death	1.1% (24/2149)	1.1% (18/1672)	n.s.
Cerebrovascular accident	2.6% (55/2099)	1.8% (30/1635)	n.s.
Major stroke	0.8% (16/2087)	0.4% (7/1627)	n.s.
Minor Stroke	0.4% (9/2087)	0.4% (6/1627)	n.s.
Periprocedural MI	0.5% (11/2096)	0.4% (6/1628)	n.s.
AKI	18.7% (301/1609)	19.2% (242/1261)	n.s.
Stage 3	0.1% (1/1609)	0.2% (2/1261)	n.s.
Major bleeding	6.5% (134/2070)	5.4% (88/1622)	n.s.
Life-threating bleeding	2.5% (51/2070)	1.4% (23/1622)	0.024
Any VC	13.7% (287/2098)	10.9% (179/1642)	0.011
Major VC	3.9% (82/2098)	2.4% (40/1642)	0.012
Repeat UI	3.3% (70/2094)	3.9% (63/1631)	n.s
Valve-in-valve Surgical revision	3.2% (67/2075) 0.1% (3/2094)	3.7% (60/1620) 0.2% (3/1631)	n.s. n.s.
Permanent PM	21.2% (410/1934)	26.1% (369/1413)	<0.001

MI = myocardial infarction; TIA = transient ischemic attack; AKI = acute kidney injury; VC = vascular complication; UI = unplanned intervention; PM = pace maker.

**Table 3:** Echocardiographic parameters after TAVR.

All Patients (*n* = 3821)			
	Female (*n* = 2149)	Male (*n* = 1672)	*p* Value
**Baseline**			
LVEF	54.3 ± 11.2	49.7 ± 12.7	< 0.001
Mean aortic gradient	52.4 ± 15.3	47.3 ± 12.8	< 0.001
Aortic regurgitation 2+	28.8	29.1	n.s.
Mitral regurgitation 2+	43.4	37.4	< 0.001
sPAP > 60 mmHg	9.4	8.2	n.s.
**Discharge**			
LVEF	55.4 ± 9.3	51.0 ± 11.4	< 0.001
Mean aortic gradient	8.7 ± 5.3	8.3 ± 4.2	n.s.
PVL 2+	11.2% (229/2046)	15.9% (252/1584)	< 0.001
Mitral regurgitation 2+	34.7% (524/1508)	29.9% (357/1194)	0.008
sPAP > 60 mmHg	3.3% (37/1107)	4.6% (39/854)	n.s.
**30-Day Follow-Up**			
LVEF	56.2 ± 9.0	52.3 ± 10.8	< 0.001
Mean aortic gradient	8.0 ± 4.2	8.1 ± 4.1	n.s.
PVL 2+	10.3% (127/1238)	16.1% (152/947)	< 0.001
Mitral regurgitation 2+	36.8% (440/1196)	30.3% (274/904)	0.002
sPAP > 60 mmHg	3.1% (32/1019)	3.6% (27/753)	n.s.

PVL = para-valvular leak.

## Data Availability

The data presented in this study are available on request from the corresponding author. The data are not publicly available due to privacy restrictions.

## Supplementary Materials

The following are available online at https://www.mdpi.com/article/10.3390/jcdd8090114/s1, Supplementary Figure S1. One-year major bleeding (MB) and stroke during the first (up to 2012) and the last (up to 2017) five years of observation in male and female, adjusted respectively for age and eGFR and age, CAD MI and PAD. Supplementary Figure S2. One-year and three-year CV death in male and female, adjusted for age, STS, previous MI and CAD.
