# Supplementary material for: Gender Differences after Transcatheter Aortic Valve Replacement (TAVR): Insights from the Italian Clinical Service Project
Source: J Cardiovasc Dev Dis. 2021 Sep 15;8(9):114. doi: 10.3390/jcdd8090114 (PMC8472227; doi:10.3390/jcdd8090114)
Supplement: Supplementary file 1 [file jcdd-08-00114-s001.zip › jcdd-1328080-supplementary.pdf]

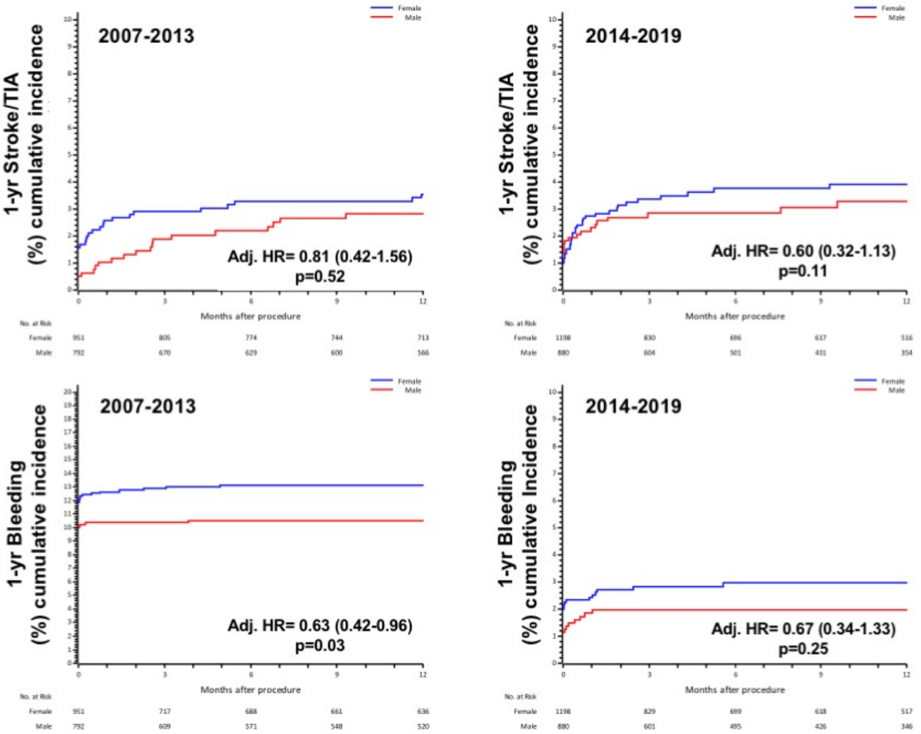

Figure S1. 1-year major bleeding (MB) and stroke during the first (up to 2012) and the last (up to 2017) five years of observation in male and female, adjusted respectively for age and eGFR and age, CAD MI and PAD.

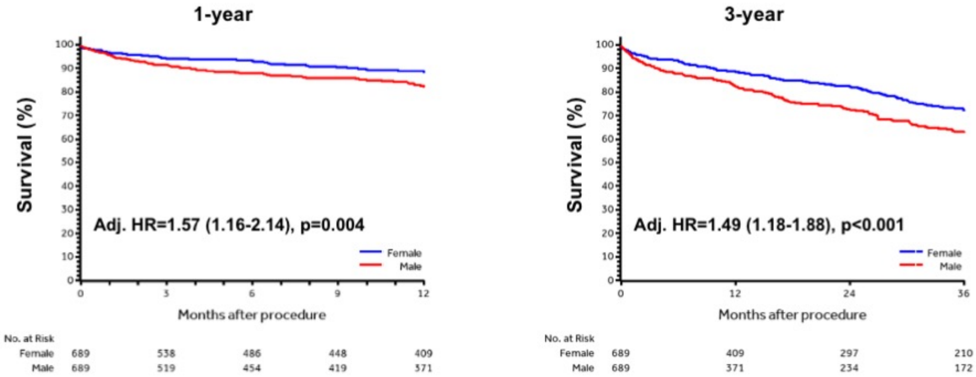

Figure S2. 1-year and 3-year death in male and female after propensity score matching.
